# Complete Genomic Sequence of *Campylobacter jejuni* subsp. *jejuni* HS:19 Strain RM1285 Isolated from Packaged Chicken

**DOI:** 10.1128/genomeA.01100-16

**Published:** 2016-10-06

**Authors:** Craig T. Parker, Steven Huynh, Astrid P. Heikema

**Affiliations:** a Produce Safety and Microbiology Research Unit, Agricultural Research Service, U.S. Department of Agriculture, Albany, California, USA; b Department of Medical Microbiology and Infectious Diseases, Erasmus MC, University Medical Center, Rotterdam, The Netherlands

## Abstract

Poultry products serve as the main source of *Campylobacter jejuni* subsp. *jejuni* infections in humans. *C. jejuni* subsp. *jejuni* infections are a leading cause of foodborne gastroenteritis and are a prevalent antecedent to Guillain-Barré syndrome. This study describes the genome of *C. jejuni* subsp. *jejuni* HS:19 strain RM1285, isolated from packaged chicken in California.

## GENOME ANNOUNCEMENT

Most *Campylobacter jejuni* subsp. *jejuni* infections are caused by the consumption of contaminated poultry products and result in an acute, self-limited gastrointestinal illness (1). In a small number of cases, *C. jejuni* subsp. *jejuni* infections are followed by the development of the autoimmune neuropathy, Guillain-Barré syndrome (GBS) (2). GBS can be elicited by *C. jejuni* sialylated lipooligosaccharides (LOS) that exhibit molecular similarity with gangliosides on human peripheral nerves (3–5). Moreover, we demonstrated previously that several capsular types, including HS:19, are markers for GBS, suggesting that capsules, located on the outer surface of *C. jejuni*, may contribute to GBS susceptibility (6). To further explore the role of capsular types in GBS, we report the first complete genomic sequence of a capsular type HS:19 *C. jejuni* subsp. *jejuni* strain, RM1285, which was isolated from packaged chicken in 1997.

Genome sequencing was performed on an Illumina MiSeq desktop sequencer using shotgun library reads. A total of 1,008,770 reads, with an average read length of 250 nucleotides, were assembled *de novo* using the Roche Newbler assembler version 2.3 and resulted in 73 total contigs (>100 bp) and 37 large contigs (5 to 77 kb). Reference assemblies against the *C. jejuni* NCTC 11168 genome (AL111168.1) and RM3196 (CP012690) were performed within Geneious software version 9.1. The *de novo* large contigs and the contigs derived from the reference assembly were used to create a draft scaffold. The scaffold gaps were filled using the small-repeat *de novo* contigs and the Perl script Contig_extender3 (7). The final genomic sequence had a coverage of 148×. Variations of the homopolymeric GC tracts were characterized within Geneious.

Protein-, rRNA- and tRNA-coding genes were identified using GLIMMER3 (8) within Geneious, RNAmmer version 1.2 (9), and tRNAscan-SE version 1.21 (10), respectively. The presence of bacteriophage-derived sequences was assessed using PHAST (11). The genomes were annotated based on those of the *C. jejuni* strains NCTC 11168 (accession no. AL111168) and RM3196 (accession no. CP012690). Additional annotations were performed using the identification of Pfam domains version 26.0 (12), and BLASTp comparisons to proteins in the NCBI nonredundant database.

The complete annotated genomic sequence of RM1285 is 1.68 Mbp and contains 1,627 open reading frames. The RM1285 genome contains an additional 35 fragmented coding sequences, identified as pseudogenes. Six flagellar modification genes and one capsular biosynthetic gene possess variable-length poly-G tracts that would result in either full-length coding sequences or pseudogenes. The annotations report the most prevalent form of the gene. A noteworthy feature possessed by this strain is the class A1 LOS locus, including a truncated version of the *cgtA* gene (annotated as a pseudogene, although it may be functional) and no poly-G tracts. Similar occurrences were found in the genomes of two GBS-related *C. jejuni* subsp. *jejuni* HS:41 strains (13). RM1285 also possesses a unique *C. jejuni* integrated element of 51 kb that is integrated into the 3′-end of a leucyl-tRNA gene.

### Accession number(s).

The whole-genome sequence and annotation were deposited in GenBank, BioProject, and BioSample under the accession numbers CP015209, PRJNA305292, and SAMN04323384.
